# CCL11 is increased in the CNS in chronic traumatic encephalopathy but not in Alzheimer’s disease

**DOI:** 10.1371/journal.pone.0185541

**Published:** 2017-09-26

**Authors:** Jonathan D. Cherry, Thor D. Stein, Yorghos Tripodis, Victor E. Alvarez, Bertrand R. Huber, Rhoda Au, Patrick T. Kiernan, Daniel H. Daneshvar, Jesse Mez, Todd M. Solomon, Michael L. Alosco, Ann C. McKee

**Affiliations:** 1 Boston University Alzheimer’s Disease and CTE Center, Boston University School of Medicine, Boston, MA, United States of America; 2 Department of Neurology, Boston University School of Medicine, Boston, MA. United States of America; 3 VA Boston Healthcare System, Boston, MA, United States of America; 4 Department of Veterans Affairs Medical Center, Bedford, MA, United States of America; 5 Department of Biostatistics, Boston University School of Public Health, Boston, MA, United States of America; 6 Framingham Heart Study, Boston University School of Medicine, Boston, MA, United States of America; 7 Department of Pathology and Laboratory Medicine, Boston University School of Medicine, Boston, MA, United States of America; Nathan S Kline Institute, UNITED STATES

## Abstract

CCL11, a protein previously associated with age-associated cognitive decline, is observed to be increased in the brain and cerebrospinal fluid (CSF) in chronic traumatic encephalopathy (CTE) compared to Alzheimer’s disease (AD). Using a cohort of 23 deceased American football players with neuropathologically verified CTE, 50 subjects with neuropathologically diagnosed AD, and 18 non-athlete controls, CCL11 was measured with ELISA in the dorsolateral frontal cortex (DLFC) and CSF. CCL11 levels were significantly increased in the DLFC in subjects with CTE (fold change = 1.234, p < 0.050) compared to non-athlete controls and AD subjects with out a history of head trauma. This increase was also seen to correlate with years of exposure to American football (β = 0.426, p = 0.048) independent of age (β = -0.046, p = 0.824). Preliminary analyses of a subset of subjects with available post-mortem CSF showed a trend for increased CCL11 among individuals with CTE (p = 0.069) mirroring the increase in the DLFC. Furthermore, an association between CSF CCL11 levels and the number of years exposed to football (β = 0.685, p = 0.040) was observed independent of age (β = -0.103, p = 0.716). Finally, a receiver operating characteristic (ROC) curve analysis demonstrated CSF CCL11 accurately distinguished CTE subjects from non-athlete controls and AD subjects (AUC = 0.839, 95% CI 0.62–1.058, p = 0.028). Overall, the current findings provide preliminary evidence that CCL11 may be a novel target for future CTE biomarker studies.

## Introduction

CTE is a progressive neurodegenerative disease associated with a history of repetitive head impacts (RHI) [1]. Over the last decade, CTE has become an increasingly recognized as a potential consequence for athletes participating in contact sports such as American football, soccer, boxing, and ice hockey, and military veterans exposed to blasts [2, 3]. A recent analysis of the brains from 111 National Football League (NFL) players observed 110 (99%) had evidence of CTE, further suggesting how prevalent the disease might be [4]. Currently, CTE can only be diagnosed by post mortem examination of brain tissue. The diagnostic hallmark of CTE is the accumulation of hyperphosphorylated tau (as neurofibrillary tangles (NFT)) in neurons and astrocytes present around small blood vessels at the sulcal depths of the cerebral cortex [5]. The development of perivascular tau pathology in CTE is associated with a robust and persistent inflammatory microglial response that significantly increases with the pathological severity of CTE [6, 7]. The pathology of CTE differs in several substantive ways from AD. AD is characterized by the accumulation of beta-amyloid (Aß) plaques as well as the deposition of aggregates of hyperphosphorylated tau (ptau) as NFTs, however in AD there is no accentuation of ptau deposition around small blood vessels and there are differences in the tau epitopes expressed in AD and CTE [8]. Although AD and CTE are both characterized by disruption of the BBB and neuroinflammation, the BBB disruption and neuroinflammation in CTE appear to predate ptau deposition [6]. A detailed analysis of the select cytokines and chemokines involved in CTE and AD has yet to be performed.

The neuropathological features of CTE have become increasingly well-defined, including recently developed diagnostic criteria [5]. However, there is an urgent need for a method to detect CTE during life. One reason that precludes that ability to diagnose CTE in life at this time is the lack of *in vivo* biomarkers that accurately detect CTE pathology. PET imaging of tau is expected to be the gold standard biomarker for CTE, however, analysis of plasma and CSF proteins is a more practical alternative that can also accurately detect the presence of neurodegenerative diseases (e.g., AD). CCL11 may be one potential candidate biomarker for CTE.

The chemokine CCL11, also known as eotaxin-1, was first identified in the peripheral immune system as a potent eosinophil chemoattractant in allergic inflammation, asthma, atopic dermatitis, and inflammatory bowel disease [9–11]. Although CCL11 has been previously observed to be produced and act mainly in the periphery, studies in mice have shown that CCL11 is capable of both penetrating the blood-brain barrier (BBB) and in aged mice, can be directly produced by the choroid plexus epithelial cells suggesting direct CNS effects [12]. Additionally, inflammatory insults have been observed to stimulate CCL11 secretion in primary cultures of astrocytes, pericytes, and microglia [13–15]. Furthermore, microglia, oligodendrocytes, astrocytes and neurons are known to express CCR1, CCR3, and CCR5, which are the cognate receptors for CCL11 [16]. These findings suggest that regardless of the production source, CCL11 is capable of reaching the central nervous system (CNS) and interacting with resident microglia, oligodendrocytes, astrocytes and neurons.

Recent studies have shown that CCL11 and related molecules play a role in neuroinflammation and neurodegeneration [17–19]. CCL11 levels increase in the plasma and cerebrospinal fluid (CSF) of mice and humans as part of normal aging [20]. In mice, these increases are associated with declining neurogenesis and impaired cognition and memory [20]. CCL11 has also been reported to enhance microglial production of reactive oxygen species and promote excitotoxic neuronal death [14]. In humans, altered CSF and plasma levels of CCL11 have been observed in Alzheimer's disease (AD), amyotrophic lateral sclerosis (ALS), Huntington's disease (HD) and secondary progressive multiple sclerosis (SPMS) when compared to age-matched, healthy controls. Notably, in AD and HD, increased plasma CCL11 expression is associated with more advanced disease, while in ALS and SPMS, lowered CCL11 expression is associated with increased disease severity [17–19, 21]. The observed differences in CCL11 expression with certain neurodegenerative diseases suggest that CCL11 is differentially regulated across disease states and may prove useful as a novel biomarker candidate to detect distinctive neurodegenerative diseases.

Using human brain tissue from several neurodegenerative disease brain banks, the purpose of this study was to compare the level of expression of CCL11 in the dorsolateral frontal cortex (DLFC) among subjects with neuropathologically verified AD, CTE and normal controls. Furthermore, preliminary analysis on CSF was performed to determine the viability of CCL11 as a potential diagnostic biomarker for CTE.

## Methods

### Subjects

Frozen brain tissue from the DLFC was obtained from 23 former male American football players with neuropathologically diagnosed CTE using recently published National Institute Neurological Diseases and Stroke (NINDS) criteria [5]. Subjects were selected from the entire 139 subjects who donated their brain and had frozen tissue available based on the following criteria: 30 subjects were excluded that did not have CTE, an additional 76 were excluded due to carrying a neuropathological diagnosis of either Alzheimer’s disease, Parkinson’s disease, Dementia with Lewey bodies, frontotemporal lobe degeneration, or motor neuron disease, lastly, 10 cases were excluded due to not playing American football. This group was designated as “CTE”. Frozen DLFC brain tissue was also obtained from Framingham Heart Study brain donors, both male and female, without a history of military exposure or participation in contact sports. This included 50 subjects with neuropathologically diagnosed AD using the National Institute on Aging–Alzheimer’s Association criteria [22] without comorbid neurodegeneration and 18 control subjects free of neurodegenerative pathology (Table 1). No statistical differences in CCL11 levels were observed between gender (Control Male vs. Control Female: p = .297, AD Male vs. AD Female: p = .272). Next of kin provided written consent for participation and brain donation. IRB approval for brain donation and this study were obtained through the Boston University Alzheimer's Disease Center (BU ADC) and CTE Center, Human Subjects Institutional Review Board of the Boston University School of Medicine, and Edith Nourse Rogers Memorial Veterans Hospital, Bedford, MA.

**Table 1:** Demographic and exposure characteristics of subject groups.

	N	Age	Years of exposure	Number of Concussions	Gender (%male)
Control	18	85.4 ± 9.4	0	0	50%
CTE	23	62.0 ± 16.5	16.0 ± 4.5	37.9 ± 53.5	100%
AD	50	83.9 ± 9.7	N/A	N/A	50%

Data expressed as mean ± SD.

### Clinical assessment

Clinical assessment occurred as previously described [4, 23]. During a telephone interview, athletic history, military service history, demographic information, and education, were assessed. An informant versions of the Ohio State University TBI Identification Method Short Form [24] and two questionnaires adapted from published studies that address military-related head injuries and concussions was used to determine TBI history [25, 26]. For analysis on how the number of years exposed to playing football relates to CCL11, subjects in the CTE group were further divided into two groups based on the group median number of years playing football (16 years). An identical clinical assessment was performed using the Framingham Heart Study cohort.

### Neuropathological examination and immunohistochemistry

Pathological processing, immunohistochemistry, and evaluation were conducted using previously published methodology [6, 27, 28]. Briefly, all brain tissue was processed identically by fixation in periodate-lysine-paraformaldehyde (PLP) and stored at 4°C. During the initial processing, macroscopic features and brain volume were recorded. Tissue was blocked and cut at 10 μm thickness. To identify the CTE specific features required for a positive neuropathologic diagnosis, 22 sections from multiple tissue regions were stained for Luxol fast blue, hematoxylin and eosin (LHE), Bielschowsky’s silver, phosphorylated tau (ptau) (AT8), alpha-synuclein (αs), amyloid-ß (Aß), and phosphorylated TDP-43 (pTDP-43) using methods described previously [29]. For histologic antibody staining, section underwent antigen retrieval using citrate buffer (pH 6.0) and boiling in the microwave for 10 mins. Primary antibodies were applied and incubated overnight at 4°C. The next day, biotinylated secondary antibodies and 3-amino-9-ethylcarbazol HRP substrate kit (Vector Laboratories H-3401) were used for visualization of staining. Sections were coverslipped for long term storage using Permount mounting medium.

As previously described [4], a neuropathological diagnosis of CTE was made using criteria recently defined by the 2015 NINDS-NIBIB Consensus Conference [5]. Other neurodegenerative diseases were diagnosed using well-established criteria for AD [30, 31], Lewy body disease (LBD) [32], frontotemporal lobar degeneration (FTLD) [33–37], and motor neuron disease (MND) [38, 39]. To diagnose CTE, the criteria requires at least one perivascular ptau lesion that consisting of ptau aggregates in neurons, astrocytes, and cell processes around a small vessel [5]. The deposition of ptau is most commonly observed at the depths of the cortical sulci in the cerebral cortex [40]. The CTE ptau deposition is distinct from the lesions of aging-related tau astrogliopathy [41].

### Microscopy and analysis

For analysis of CTE pathologic severity, AT8 (ptau) immunostained slides from the DLFC were scanned and digitized at 20x magnification using the Aperio ScanScope (Leica) as previously described [42]. Identification, selection, and analysis of the regions of interest were performed as previously described [6]. Briefly, the depth of the cortical sulcus, which was defined as the bottom third of two connecting gyri, was selected and circled in ImageScope (Lecia). Only gray matter was highlighted. A modified version of the Aperio positive pixel count algorithm (Version 9) was used to determine the total area of AT8 positive staining. Quantifications were standardized to the area measured and presented as density per analyzed area as previously described [6].

### Enzyme-Linked Immunosorbent Assay (ELISA)

Flash frozen brain tissue was obtained from the sulcus of the DLFC, weighed, and placed on dry ice. Freshly prepared, ice cold 5M Guanidine Hydrochloride in Tris-buffered saline (20 mM Tris-HCl, 150 mM NaCl, pH 7.4 TBS) containing 1:100 Halt protease inhibitor cocktail (Thermo Scientific) and 1:100 Phosphatase inhibitor cocktail 2 & 3 (Sigma) was added to the brain tissue at 5:1 and homogenized with Qiagen Tissue Lyser LT, at 50Hz for 5 minutes. The homogenate was then incubated while rocking overnight at room temperature. Lysate was diluted according to manufacture protocol and spun down at 17,000 g, 4°C, for 15 minutes. The supernatant was then applied to Meso Scale Discovery (MSD) Chemokine Panel 1 (human) Kit V-PLEX Plus (Thermo Scientific) and run according to manufactures protocol. Plates were run on MSD plate reader model 1250. Plates were run in three separate batches. In order to account for inter-batch variability, values were normalized to controls present on each respective batch to generate a “fold change” value compared to the controls.

Post-mortem cerebrospinal fluid (CSF) was obtained from the foramen magnum by gently lifting the frontal lobes to access with a large bore needle. CSF was then mixed by gently inverting the tube 5 times. The tubes were centrifuged at 1.5 g for 15 minutes at 4°C. The CSF supernatant was carefully removed with a transfer pipet, leaving about 300 μL in the bottom, and aliquoted into 1.5 mL microcentrifuge polypropylene tubes. CSF was stored at -80°C prior to use. CSF was then run undiluted using R&D Quantikine Human CCL11/CCL11 ELISA according to manufactures protocol. Plates were imaged using a SpretraMax M3 imager (Molecular Devices). CSF was available from only a small group of cases: 4 controls, 7 CTE, and 4 AD. Values were standardized against the controls to generate relative fold change.

### Statistics

Statistical analysis was performed using SPSS (v.24; IBM, Inc., Armonk, NY).and Prism (v. 6,Graphpad Software, La Jolla, CA). AT8 density and the number of reported concussions were log transformed to normalize for regression analysis. A one-way analysis of covariance (ANOVA) was used to compare CCL11 fold changes among control, CTE, and AD groups. Age at death was included in all regressions analyses to control for age-associated changes. Separate multiple linear regression analyses were used to compare CCL11 expression levels to AT8 tau density, years of exposure, and number of concussions. Binary logistic regressions were used to determine the association between CCL11 and CTE or AD. Receiver operating characteristic (ROC) curve analysis was used to determine sensitivity and specificity of CCL11 as a biomarker that can predict CTE in between controls, CTE, and AD cases.

Descriptive statistics were generated using SPSS (v.24; IBM, Inc., Armonk, NY).

## Results

### CCL11 is elevated in CTE

**Fig 1:**
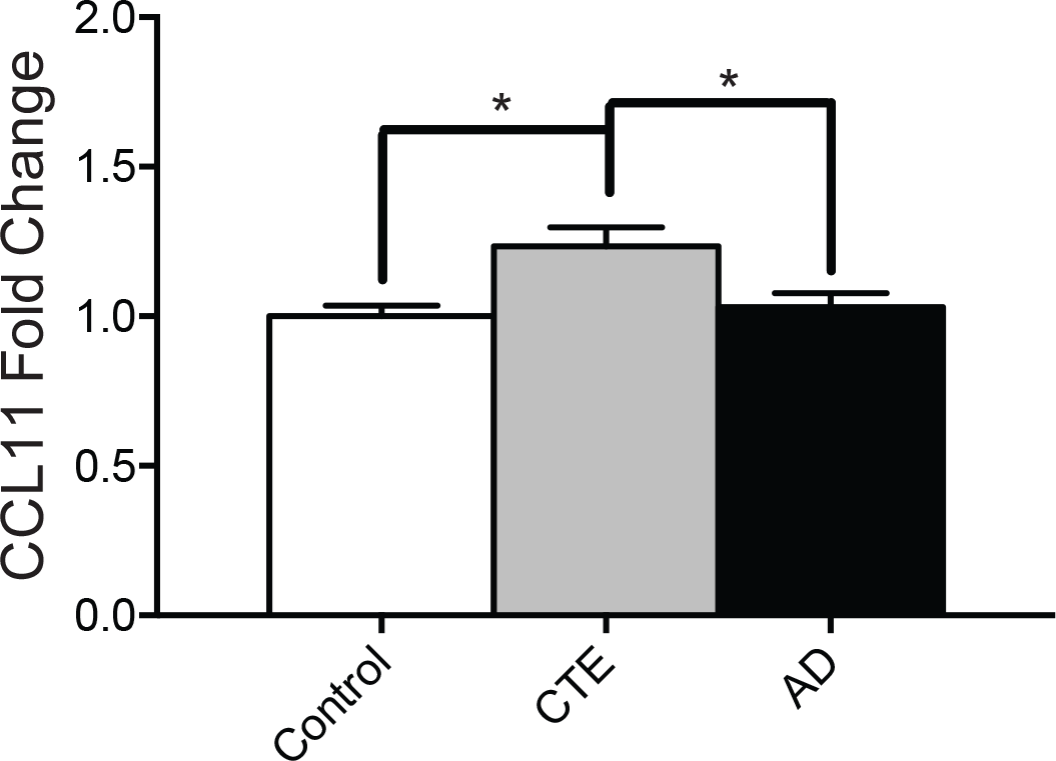
Protein levels of CCL11 are elevated in the DLFC in CTE but not AD. CCL11 protein levels were measured using ELISA. CCL11 fold change is shown for non-exposed control, CTE, and AD subjects. Bar graphs shows mean ± SEM, *p < 0.05; One-way ANOVA.

Total levels of CCL11 were significantly increased in subjects with CTE compared to controls or to subjects with AD as measured by ELISA (Fig 1). Furthermore, when pooling the control, AD, and CTE subjects together, a binary logistic regression was able to significantly discriminate a positive neuropathologic diagnosis of CTE when using CCL11 fold changes (OR = 76.382, p = 0.024), independently of age (OR = 0.850, p = 0.001) and gender (OR < 0.001, p = 0.997). In contrast, binary logistic regression was not able to discriminate between a positive or negative neuropathologic diagnosis of AD using CCL11 levels (OR = 0.394, p = 0.238) when controlling for age (OR = 1.053, p = 0.009) and gender (OR = 2.324, p = 0.111). In subjects with CTE, multiple linear regression also demonstrated that AT8 tau density at the sulcal depths of the DLFC is significantly predicted by CCL11 levels (β = 0.430, p = 0.035) independent of age (β = 0.404, p = 0.047).

### Years of exposure to American football is associated with greater CCL11 levels

**Fig 2:**
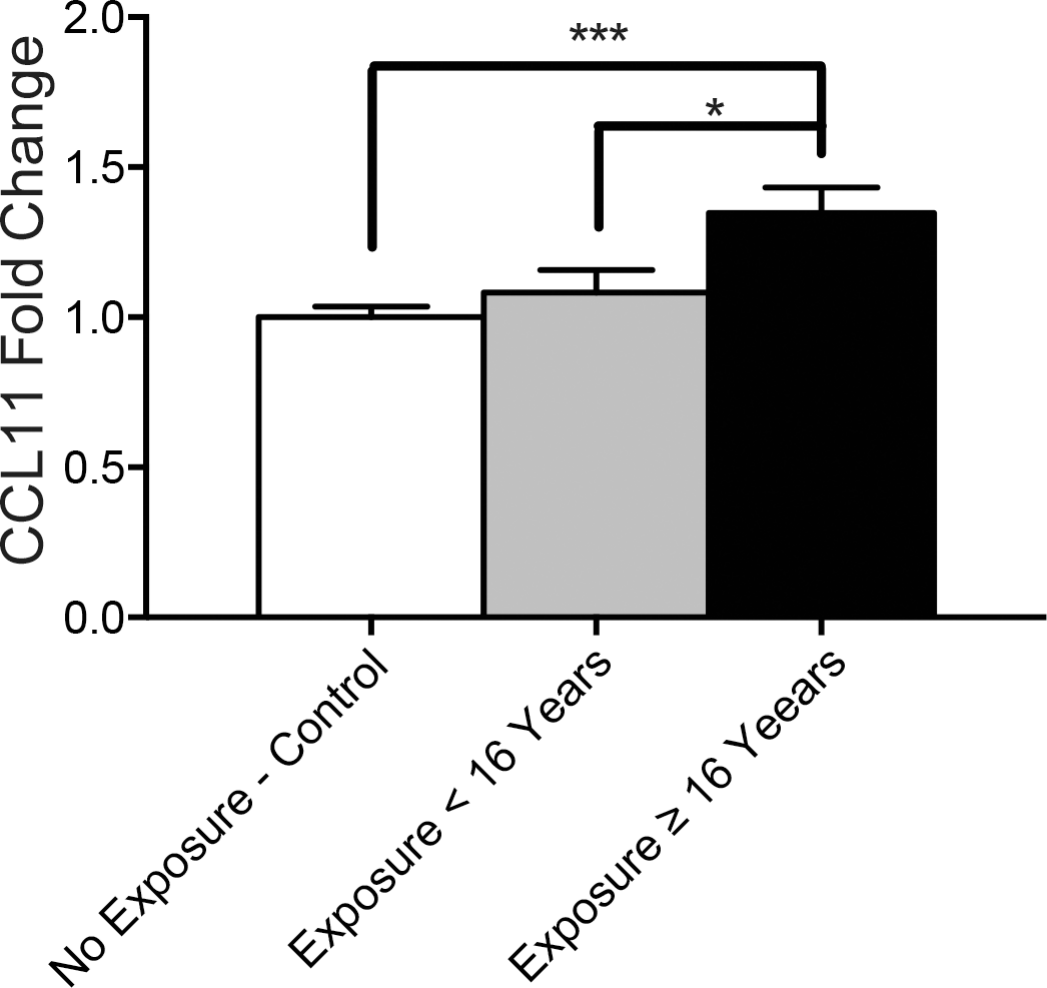
CCL11 is elevated in cases with more than 16 years of exposure to American football. CCL11 protein fold changes are shown for non-exposed controls (n = 18), cases with less than 16 years of exposure (n = 10), and cases with more than or equal to 16 years of exposure (n = 13). Bar graphs shows mean ± SEM, *p < 0.05, ***p < 0.001; One-way ANOVA.

A significant increase in CCL11 levels was observed in individuals with CTE and 16 years or more exposure to football compared to controls with no exposure to sports and individuals with CTE and less than 16 years exposure (Fig 2). Furthermore when looking only at individuals with CTE, multiple linear regression analysis demonstrated that CCL11 levels were significantly predicted by the number of years of exposure to football (β = 0.426, p = 0.048) independent of age (β = -0.046, p = 0.824). The number of reported concussions were not able to predict CCL11 levels (β = -0.230, p = 0.357) independently of age (β = -0.128, p = 0.605)

### CCL11 in the cerebrospinal fluid (CSF) in CTE

**Fig 3:**
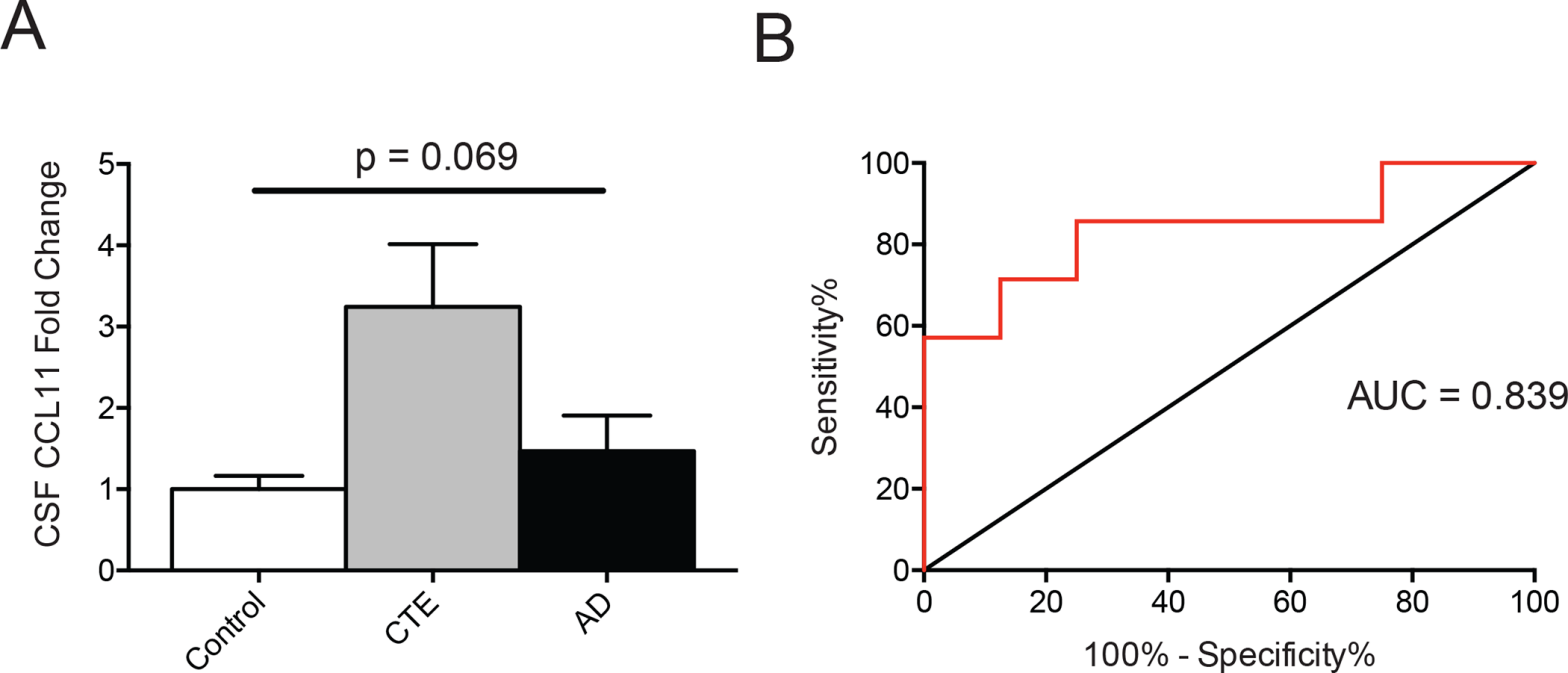
CCL11 is elevated in the CSF during CTE. (A) Quantitation of CCL11 fold change in the CSF is shown for control (n = 4), CTE (n = 7), and AD (n = 4) subjects. (B) Receiver operatic characteristic (ROC) curve for CSF CCL11 predicting CTE. Red line denotes CCL11 while the black line is the reference, AUC = 0.839, 95% CI 0.62–1.058, p = 0.028. Bar graphs shows mean ± SEM, One-way ANOVA.

In preliminary analysis using a small cohort of cases with available post-mortem CSF (Control = 4, CTE = 7, AD = 4), there was a trend towards increased CCL11 levels in CTE, but not AD, compared to controls (Fig 3A). Multiple linear regression analysis also demonstrated that CCL11 levels in the CSF were associated with greater exposure to RHI (β = 0.685, p = 0.040) independent of age (β = -0.103, p = 0.716). Furthermore, despite the limited sample size, receiver operating characteristic (ROC) curve analysis demonstrated that CSF CCL11 levels significantly discriminated participants with CTE from controls and individuals with AD (AUC = 0.839, 95% CI 0.62–1.058, p = 0.028) (Fig 3B). A cutoff value of 5.630 pg/ml was determined to have a maximum sensitivity and specificity of 71.43% and 87.5% respectively. Finally, although CSF CCL11 could significantly predict a diagnosis of CTE (Fig 3B), CCL11 levels in the CSF were not able to predict severity of disease as measured by AT8 density in the DLFC (β = 0.424, p = 0.247).

## Discussion

Overall, preliminary analysis demonstrated that levels of CCL11 were significantly elevated in the DLFC of former American football players with CTE compared to controls and individuals with AD without exposure to football. Furthermore, in individuals with CTE, CCL11 levels correlated with increased density of AT8 immunopositive tau pathology in the DLFC independently of age. Additionally, greater total number of years of football participation was significantly associated increased levels of CCL11 in subjects with CTE. Finally, CCL11 was increased in the CSF of participants with CTE and significantly predicted a positive diagnosis of CTE when compared to the CSF of controls and individuals with AD. Due to the limited sample size, this study was most likely too underpowered to observe an effect of CSF CCL11 on CTE severity. Although additional studies are needed, these preliminary data suggest that CCL11 may be a novel biomarker to aid in the detection of CTE neuropathology and to discriminate CTE from AD.

There are several limitations to this preliminary study. Age is an important component of neurodegeneration and has been hypothesized to be critical in age related neurocognitive changes [20, 43]. The mean age of the CTE cohorts was several years younger than both the control and AD cohort. To account for this, age at death was included into the regression analysis; however, the difference still remains as a potential confounding factor and future analyses will require more comparable age ranges between cohorts. Additionally, contrary to recent publications [20], age related increases in CCL11 were not observed. In CTE subjects, it was found that tau pathology in the DLFC and years of exposure to RHI were more predictive than age. There are several explanations for this. The study performed by Villeda et al. demonstrated significant plasma and CSF CCL11 differences between those within an age range of 20–45 years old and those 65–90 [20]. The majority of the subjects presented here were over the age of 50, which likely prevented proper observations of the full spectrum of CCL11 concentrations as this study does not capture the CCL11 levels in younger cases. Additional studies will be needed to determine if similar CCL11 dynamics are present in younger cases after RHI.

An additional explanation for the lack of age-associated correlations is that the years of RHI and the subsequent neuroinflammation mimics the effects of aging resulting in an environment similar to advanced aging. This could result in the neuroinflammatory environment of young and old individuals to appear more similar and obscure potential age related changes. Neuroinflammation is part of the normal aging process and has been termed “inflammaging” [44]. CCL11 is believed to be part of this inflammaging process. Previous work has demonstrated that those exposed to RHI, even at a young age, have significant chronic neuroinflammation compared to older controls [6]. By creating an inflammatory environment similar to advanced aging, CCL11 concentrations could be higher, even in younger cases. This would make years of RHI a more significant predictor of CCL11 as opposed to age, as is seen in this study.

There are other limitations as well. In order to increase the power of this preliminary study, individuals of both genders were included into the control and AD cohorts in order to maximize the chances of observing significant results. Although, no significant CCL11 changes were observed between genders, neuropathological gender differences have been reported in the past [45]. Future validation studies with more subjects will be needed to exclude gender related differences in biomarkers after head trauma. Additionally, there is selection bias in an autopsy-based study of individuals whose brains are donated by the family, and the subjects may not represent the population as a whole. Clinical and RHI exposure histories are obtained retrospectively and are subject to bias. Lastly, the CSF analysis was limited by the low sample size. Additional subjects with postmortem and antemortem CSF will be required to confirm and extend these results.

Clinically, CTE and AD may be similar in their clinical presentation [46]. Therefore, biomarkers will be necessary to differentiate these diseases in life. Previous studies have shown that CCL11 was elevated in the serum of individuals with AD but observed no changes in CSF [21, 47]. In agreement, this study found no increase in CCL11 levels within the CSF of AD subjects. However, there was an increase in CTE subjects (Fig 3A). Furthermore, a ROC curve demonstrated CCL11 was both specific and sensitive enough to distinguish a CTE diagnosis from controls or those with AD (Fig 3B). If similar results are found in antemortem CSF from a larger number of subjects, CCL11 may be a helpful biomarker for discriminating CTE from control and AD subjects. It will also be important to determine whether there is any relationship between serum CCL11 levels and levels in the CSF and brain.

The reason for the difference in CCL11 levels between AD and CTE subjects is unclear. However, it could be related to the cellular source in the brain. The choroid plexus (CP) has been suggested to be the a possible CNS endogenous CCL11 source [12]. Epithelial CCL11 production in the CP has been previously shown to be dependent on the ratio of the cytokines IFNγ and IL-4 [12]. A murine model of aging observed that higher CP mRNA levels of IL-4 correlated with higher CCL11 levels while the opposite was observed for IFNγ [12] suggesting this ratio could control CCL11 production. Interestingly, brain IFNγ levels have been shown to be elevated in AD [48] suggesting that IFNγ may keep CCL11 levels lower in AD brain tissue, but not blood. Additionally, the CP is an area that is not typically believed to be involved in early AD while TBI has been shown to lead to CP damage and immune cell entry into the brain. It has been hypothesized that the CP is an entry point for neutrophils and other peripheral immune cells post TBI [49]. Furthermore, the CP has been demonstrated to produce chemokines in response to TBI [50]. This suggests that CCL11, as well as other important chemokines, could be produced as an acute phase neuroimmune response post TBI. Just as neuroinflammation is initially a protective response [51], initial CP CCL11 signaling might be needed to recruit a repair response after TBI. However, many years of RHI may lead to chronic increases in CCL11 and detrimental downstream effects. In addition to the CP, the endogenous microglia and astrocytes could also contribute to CCL11 production post TBI and be involved in observed difference between AD and CTE. Previous reports have demonstrated glial neuroinflammation and potentially cytokine production is an early event in CTE pathogenesis [6]. However, while both astrocytes and microglia have been observed to produce CCL11 [14, 15], additional studies are needed to determine if the unique environmental factors surrounding AD and CTE (i.e. aging vs. head trauma) would contribute to the differential expression during disease.

There are several hypotheses on how CCL11 exerts its effect in the CNS. Microglia [52], astrocytes [52], and neurons [53, 54] all express the CCL11 receptor, CCR3, demonstrating CCL11 can affect a wide variety of CNS targets. Several studies have suggested CCL11 ultimately plays a role in cognitive impairment [12, 20] and therefore, either directly or indirectly, affect neurons. To that end, CCL11 has been shown to both impair neurogenesis [20] and reduce synaptic density in mouse models [53]. CCL11 has also been found to recruit microglia and increase reactive oxygen production [14]. The subsequent neuroinflammation and resulting synaptic dysfunction or neuronal death might contribute to the tau pathology in CTE and lead to cognitive impairment [6].

Several promising targets for biomarkers have been proposed for the in vivo detection of CTE. For instance, MRI based techniques such as diffusion tensor imaging (DTI) have been used to identify axonal injury and white matter abnormalities, which have been related to CTE progression [55]. Additionally, PET imaging has been developed to observe pathologic proteins such as p-tau in living brains [56]. Fluid biomarkers targeting substances in the CSF or peripheral blood have also been examined [57]. Peripheral blood represents an attractive target for biomarkers due to relative ease of obtaining from living individuals and the increased quantity compared to the CSF. Several promising blood based targets have been identified including, total tau and exosomal tau [58–60]. While both the blood and CSF can reflect changes occurring in the brain, the CSF is of special interest due its mechanistic function and dynamic relationship with the brain. The CSF is in continuity with the interstitial space and plays a role in the glymphatic clearance of solutes [61]. Thus, it may be an important compartment to identify neuropathological changes and several CSF biomarkers are already in development. Tau and neurofilament light chain (NF-L) [62] have been used as potential measures of axonal injury while S100β and GFAP have been reported to be elevated following contact sports play [63]. Ultimately, no one biomarker may be adequate for proper identification of CTE. The results of the current study used subjects with AD or CTE in the absence of any other co-neurodegenerative disease. However, amyloid beta pathology is observed in 52% of CTE cases [64] and a diagnosis of CTE with comorbid AD can occur, which can complicate biomarker interpretation. Additionally, several other diseases like ALS, Lewey body dementia, or frontotemporal lobe degeneration can exist as copathologies with CTE. CCL11 will likely be most useful as one biomarker in a panel of multiple biomarkers that can capture multiple aspects of a diverse range of neuropathologies. This suggests that utilizing CCL11 in combination with imaging biomarkers like amyloid and tau PET scans, in addition to the many fluid biomarkers would be ideal for the most sensitive and specific diagnostics of CTE. The preliminary data presented here, describes CCL11 as another potential biomarker to aid in clinical diagnosis of CTE and to help discriminate from other neurodegenerative diseases such as AD.

Overall, CCL11 levels in the brain were selectively increased in a group of CTE subjects compared to control or AD subjects without a history of RHI. Similar changes were seen in a preliminary analysis of postmortem CSF from a subset of subjects. CCL11 may be a potential diagnostic biomarker useful in life. Future studies are necessary to determine whether CCL11 is an early or late change in CTE and whether levels are predictive of clinical course.
